# Occlusive retinal vasculitis and scleritis following brolucizumab treatment: A case report

**DOI:** 10.1097/MD.0000000000040154

**Published:** 2024-10-18

**Authors:** Shunpei Onagawa, Masahiro Miura

**Affiliations:** a Department of Ophthalmology, Tokyo Medical University, Ibaraki Medical Center, Inashiki, Ibaraki, Japan.

**Keywords:** brolucizumab, case report, complication, occlusive retinal vasculitis, scleritis

## Abstract

**Rationale:**

Brolucizumab is an anti-vascular endothelial growth factor agent. Clinical trials have demonstrated excellent efficacy of brolucizumab for neovascular age-related macular degeneration in terms of both visual and anatomic outcomes. However, compared with conventional anti-vascular endothelial growth factor therapy, this new treatment has a higher incidence of complications, particularly the development of occlusive retinal vasculitis. In this case report, we describe a patient who developed occlusive retinal vasculitis following brolucizumab treatment for age-related macular degeneration, followed by scleritis 141 days later.

**Patient concerns:**

A 67-year-old Japanese man with a diagnosis of polypoidal choroidal vasculopathy in his right eye has received 18 intravitreal injections of aflibercept in the past 4 years. Because of a decline in treatment efficacy, intravitreal brolucizumab injection (IVBr) was initiated. However, 17 days after the second IVBr, the patient developed extensive occlusive retinal vasculitis with intraocular inflammation.

**Diagnosis:**

Occlusive retinal vasculitis in the right eye was diagnosed as a complication of brolucizumab therapy.

**Interventions:**

Corticosteroid treatment was initiated.

**Outcomes:**

The occlusive retinal vasculitis resolved 121 days after the second IVBr, and corticosteroid treatment was discontinued on day 138. However, on day 158 after the second IVBr, scleritis with intraocular inflammation developed. By day 184 after the second IVBr, both the scleritis and intraocular inflammation had resolved with the resumption of topical corticosteroid treatment.

**Lessons:**

This case underscores the potential for brolucizumab-induced scleritis and emphasizes the importance of recognizing and promptly managing this complication. Furthermore, it highlights the need for long-term careful follow-up in patients who develop occlusive retinal vasculitis after brolucizumab treatment.

## 1. Introduction

Brolucizumab (Beovu; Novartis, Basel, Switzerland) is a next-generation anti-vascular endothelial growth factor therapy that inhibits all vascular endothelial growth factor-A isoforms. In 2 parallel phase 3 randomized clinical trials—HAWK and HARRIER—brolucizumab demonstrated improved tissue penetration, a greater drying effect, and prolonged durability for neovascular age-related macular degeneration (AMD) compared with aflibercept.^[1]^ Based on the results of these clinical trials, brolucizumab was approved as a treatment for neovascular AMD in various countries, including the United States and Japan.

Since this approval, however, there have been post-marketing reports of retinal vasculitis, including occlusive retinal vasculitis, typically occurring in the presence of intraocular inflammation. A post hoc review of the HAWK and HARRIER studies by the safety review committee^[2]^ revealed an overall incidence of 4.6% for intraocular inflammation, 3.3% for intraocular inflammation with retinal vasculitis, and 2.1% for intraocular inflammation with retinal vasculitis and occlusive retinal vasculitis.^[2]^ Intraocular inflammation associated with a loss of 15 or more letters of visual acuity at the last visit or the end of the study occurred in 0.7% of patients.^[2]^ Subsequent reports have described additional complications associated with brolucizumab treatment, including scleritis^[3]^ and marked choroidal thinning.^[4]^

In this case report, we describe a patient who developed occlusive retinal vasculitis following brolucizumab treatment for AMD, followed by scleritis 141 days later.

## 2. Case report

A 67-year-old Japanese man with a diagnosis of polypoidal choroidal vasculopathy in his right eye has received 18 intravitreal injections of aflibercept in the past 4 years. As the therapeutic efficacy gradually decreased, his best-corrected visual acuity (BCVA) declined from 1.2 to 0.5. The patient had no history of autoimmune or inflammatory conditions, and his right eye exhibited mild cataract formation with no other ocular pathology. Color fundus photography and optical coherence tomography revealed hemorrhagic pigment epithelial detachment in the right eye. Given the declining BCVA, the decision was made to initiate treatment with brolucizumab (6 mg/0.05 mL, Beovu; Novartis) as a novel approach for managing his condition. Before the first intravitreal brolucizumab injection (IVBr), the BCVA in the patient’s right eye was 0.5. He received a second IVBr 53 days after the first. On ophthalmological examination at 13 days after the second IVBr (66 days after the first IVBr), no treatment-related complications, including retinal vasculitis and endophthalmitis, were observed.

**Figure 1.**
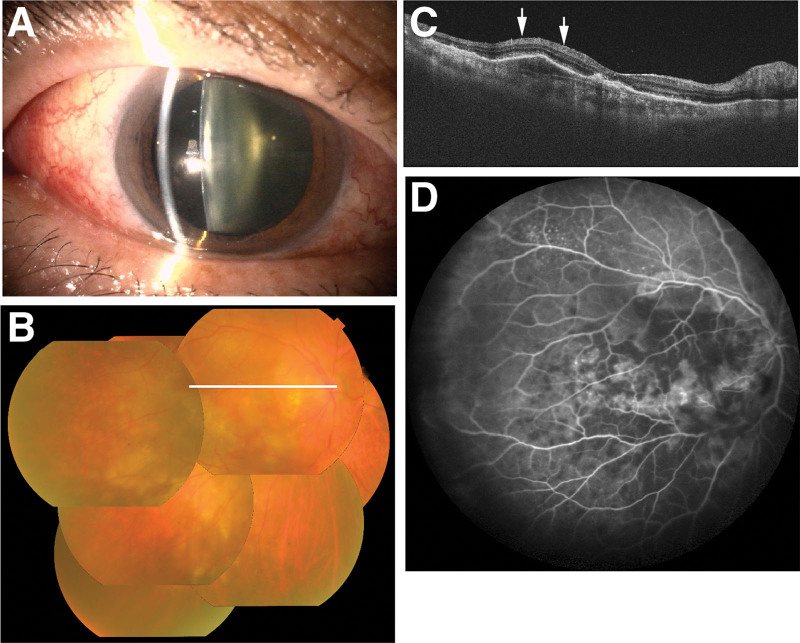
Occlusive retinal vasculitis with intraocular inflammation on day 17 after the second brolucizumab treatment. (A) Slit lamp microscopy photograph showed inflammation in the anterior chamber and vitreous body, along with mild ciliary congestion. (B) Color fundus photography showed retinal whitening with multiple cotton wool spots. The white line in this photograph indicates the scanning line of optical coherence tomography. (C) Optical coherence tomography showed hyper-reflectivity of the inner retinal layer secondary to retinal ischemia (white arrows). (D) Fundus fluorescein angiography demonstrated retinal artery occlusion with vascular leakage.

However, on day 17 after the second IVBr (70 days after the first IVBr), the patient experienced visual disturbance and ocular pain in his right eye. His BCVA declined to 0.06. Slit lamp examination revealed inflammation in the anterior chamber and vitreous body, along with mild ciliary congestion (without scleritis) (Fig. 1A). Color fundus photography showed retinal whitening with multiple cotton wool spots (Fig. 1B). Optical coherence tomography showed hyper-reflectivity of the inner retinal layer secondary to retinal ischemia (Fig. 1C). Fundus fluorescein angiography demonstrated retinal artery occlusion with vascular leakage (Fig. 1D). Based on these findings, the patient was diagnosed with extensive occlusive retinal vasculitis and intraocular inflammation. Presuming this to be a complication of IVBr, corticosteroid treatment was initiated. The patient received oral prednisolone at 20 mg/d, topical 0.01% betamethasone sodium phosphate 6 times daily, and a 20-mg triamcinolone acetonide injection into the sub-Tenon’s capsule. The inflammation in the anterior chamber and vitreous body resolved by day 95 after the second IVBr (148 days after the first IVBr), but the vitreous opacity remained. The occlusive retinal vasculitis improved by day 121 after the second IVBr (174 days after the first IVBr). The corticosteroid treatment was subsequently discontinued on day 138 after the second IVBr (191 days after the first IVBr), and the BCVA in the right eye improved to 0.08. The posterior subcapsular cataracts gradually progressed.

**Figure 2.**
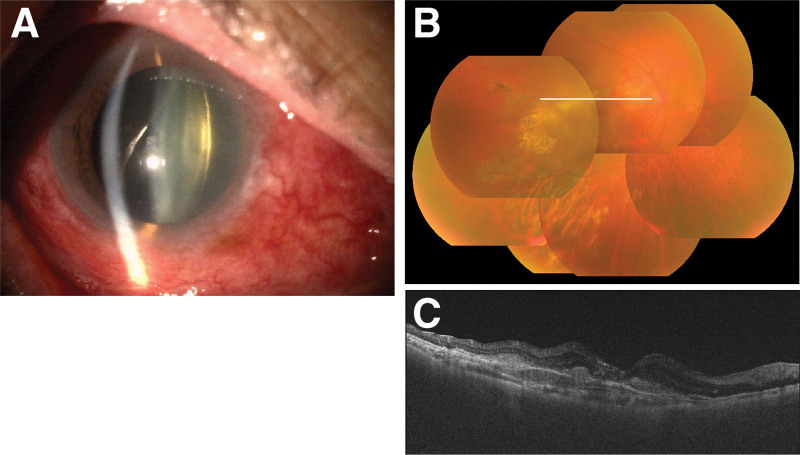
Scleritis with intraocular inflammation on day 158 after the second brolucizumab treatment. (A) Slit lamp microscopy photograph showed scleritis with inflammation in the anterior chamber. (B) The white line in the color fundus photograph indicates the scanning line of optical coherence tomography. Neither the (B) color fundus photograph nor (C) optical coherence tomography showed reemergence of the occlusive retinal vasculitis. (C) The optical coherence tomography image was somewhat blurred by anterior chamber inflammation and vitreous opacity.

**Figure 3.**
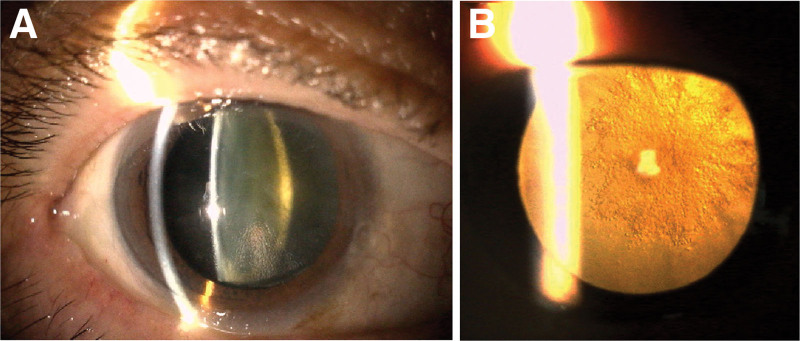
Slit lamp microscopy photographs on day 184 after the second brolucizumab treatment. (A) The scleritis and anterior chamber inflammation had resolved, but (B) the posterior subcapsular cataracts had progressed.

However, on day 158 after the second IVBr (211 days after the first IVBr), the patient again experienced visual disturbance and ocular pain in his right eye. His BCVA dropped to 0.02. Slit lamp examination revealed scleritis with inflammation in the anterior chamber (Fig. 2A). Interestingly, there was no re-emergence of the occlusive retinal vasculitis (Fig. 2B, C). Topical 0.01% betamethasone sodium phosphate was reinitiated 6 times daily. By day 184 after the second IVBr (237 days after the first IVBr), the scleritis and anterior chamber inflammation had resolved, but the posterior subcapsular cataract had worsened (Fig. 3). On day 279 after the second IVBr (332 days after the first IVBr), the corticosteroid therapy was tapered down to topical 0.1% fluorometholone, and cataract surgery was carefully planned. The BCVA in the patient’s right eye was 0.03.

Throughout the follow-up period, the intraocular pressure in the right eye remained within the normal range of 12 to 19 mm Hg. Blood tests and chest radiography were performed to investigate the cause of the scleritis and occlusive retinal vasculitis, but no evidence of autoimmune diseases such as rheumatoid arthritis, anti-neutrophil cytoplasmic antibody-associated vasculitis, or systemic lupus erythematosus was identified.

## 2.1. Ethics statement

Ethical approval for this study was obtained from the Institutional Review Board of Tokyo Medical University (approval number: T2020-142). The study protocol adhered to the tenets of the Declaration of Helsinki. Written informed consent for the publication of this case report, including the accompanying images, was obtained from the patient after providing him with a detailed explanation of the report and ensuring his full understanding.

## 3. Discussion

Takayama et al^[3]^ reported 3 cases of scleritis associated with brolucizumab treatment for AMD. Initially, they did not recognize scleritis as a complication of brolucizumab treatment and continued IVBr in 2 cases after the onset of scleritis. Consequently, 1 eye developed occlusive retinal vasculitis. In our case, intraocular inflammation with occlusive retinal vasculitis was observed 17 days after the last brolucizumab treatment, followed by the development of scleritis 141 days later. Neither our patient nor the 3 patients described by Takayama et al^[3]^ had a history of scleritis or any underlying diseases associated with it. Furthermore, our patient developed scleritis after discontinuing corticosteroid treatment for occlusive retinal vasculitis, suggesting that scleritis may be part of a spectrum of intraocular inflammation induced by brolucizumab treatment. Considering these findings, we propose that scleritis should be recognized as a potential complication of brolucizumab treatment.

In our patient, corticosteroid treatment was discontinued after 121 days because of the resolution of intraocular inflammation with occlusive retinal vasculitis. However, scleritis developed 20 days later. The average time to resolution of intraocular inflammation with occlusive retinal vasculitis is reportedly 98 days, which is significantly longer than the average of 54 days for intraocular inflammation alone.^[5]^ This suggests that in our case, because of the presence of occlusive retinal vasculitis, the inflammation took longer to resolve and scleritis developed even after long-term corticosteroid treatment. Therefore, careful long-term follow-up is necessary for patients who develop occlusive retinal vasculitis after brolucizumab treatment.

Additionally, our patient developed a posterior subcapsular cataract. The cataract may have been a complication of intraocular inflammation^[6]^ or a side effect of corticosteroid treatment.^[7]^ In either case, it was related to the complications of brolucizumab treatment and can be considered a broad-sense complication. At the time of this writing, the patient’s corticosteroid treatment was being cautiously continued and gradually tapered with a plan to perform cataract surgery after corticosteroid discontinuation.

This case report has several limitations. Our report is limited to a single case, and a definitive conclusion regarding the association of brolucizumab treatment with occlusive retinal vasculitis and scleritis cannot be drawn. Furthermore, including our case, only 4 cases of scleritis following brolucizumab treatment have been reported,^[3]^ and much remains unknown regarding the causative factors and treatment strategies. Although corticosteroid treatment is recommended for managing complications of brolucizumab treatment,^[5]^ a definitive treatment strategy for individual cases has not yet been established. Further research with larger numbers of cases is warranted.

In summary, this case underscores the potential for brolucizumab-induced scleritis and emphasizes the importance of recognizing and promptly managing this complication. Ophthalmologists should vigilantly monitor patients receiving brolucizumab therapy for any signs of scleritis and consider discontinuing brolucizumab if scleritis develops. Early intervention with appropriate anti-inflammatory therapy can help prevent further visual deterioration and complications. Our case also highlights the need for long-term follow-up in patients who develop occlusive retinal vasculitis after brolucizumab treatment. Although the resolution of intraocular inflammation might suggest clinical improvement, the potential for delayed scleritis necessitates ongoing monitoring. Ophthalmologists should exercise continued vigilance in assessing patients for signs of scleritis, even after the resolution of other inflammatory manifestations.

## Acknowledgments

We thank Angela Morben, DVM, ELS, from Edanz (https://jp.edanz.com/ac) for editing the draft of this manuscript.

## Author contributions

**Conceptualization:** Shunpei Onagawa, Masahiro Miura.

**Formal analysis:** Shunpei Onagawa, Masahiro Miura.

**Investigation:** Shunpei Onagawa, Masahiro Miura.

**Project administration:** Shunpei Onagawa, Masahiro Miura.

**Validation:** Shunpei Onagawa, Masahiro Miura.

**Visualization:** Shunpei Onagawa, Masahiro Miura.

**Writing – original draft:** Shunpei Onagawa, Masahiro Miura.

**Writing – review & editing:** Shunpei Onagawa, Masahiro Miura.

**Data curation:** Masahiro Miura.

**Funding acquisition:** Masahiro Miura.

**Supervision:** Masahiro Miura.
